# No impact of nitrogen fertilization on carbon sequestration in a temperate *Pinus densiflora* forest

**DOI:** 10.1038/s41598-023-27989-3

**Published:** 2023-03-06

**Authors:** Gyeongwon Baek, Hyungwoo Lim, Nam Jin Noh, Choonsig Kim

**Affiliations:** 1 Division of Environmental and Forest Science, Gyeongsang National University, Jinju, 52725 South Korea; 2 Department of Forest Ecology and Management, Swedish University of Agricultural Sciences, 901 83 Umeå, Sweden; 3 Institute of Ecology and Earth Sciences, University of Tartu, 50409 Tartu, Estonia; 4 Department of Forest Resources, Kangwon National University, Chuncheon, 24341 South Korea

**Keywords:** Forest ecology, Forestry, Ecophysiology

## Abstract

Carbon (C) sequestration capacity in forest ecosystems is generally constrained by soil nitrogen (N) availability. Consequently, N fertilization is seen as a promising tool for enhancing ecosystem-level C sequestration in N-limited forests. We examined the responses of ecosystem C (vegetation and soil) and soil N dynamics to 3 years of annual nitrogen-phosphorus-potassium (N_3_P_4_K_1_ = 11.3 g N, 15.0 g P, 3.7 g K m^−2^ year^−1^) or PK fertilization (P_4_K_1_), observed over 4 years in a 40-year-old *Pinus densiflora* forest with poor N nutrition in South Korea. PK fertilization without N was performed to test for PK limitation other than N. Neither tree growth nor soil C fluxes responded to annual NPK or PK fertilization despite an increase in soil mineral N fluxes following NPK fertilization. NPK fertilization increased the rate of N immobilization and 80% of the added N was recovered from mineral soil in the 0–5 cm layer, suggesting that relatively little of the added N was available to trees. These results indicate that N fertilization does not always enhance C sequestration even in forests with poor N nutrition and should therefore be applied with caution.

## Introduction

It is widely accepted that carbon (C) sequestration capacity in temperate and boreal forest ecosystems is constrained by soil nitrogen (N) availability^1–6^. Therefore, N fertilization can be used to increase forest C sequestration^7,8^. The ecosystem C response to N, however, depends strongly on N nutrition of trees in the forest; the greater the foliar N concentration of the trees, the smaller the increase in C sequestration following N fertilization^9^. Forests suitable for N fertilization can therefore be identified by measuring the foliar N concentration. The optimal foliar N concentration for conifer trees is between 1.5 and 2.5%; values below this range are associated with N limitation, whereas higher values lead to nutrient imbalances that adversely affect forest health and production^10–15^.

Adding fertilizer to a nutrient-limited system enhances ecosystem C sequestration, primarily by increasing tree growth (17–25 g C sequestered g^−1^ N added) but also to a lesser extent by promoting soil C accumulation (11–23 g C g^−1^ N)^16^. The N response of tree growth is associated with increased canopy C assimilation and increased C partitioning to woody biomass^7,17–19^. The soil C accumulation response to N fertilization occurs primarily in the organic layer^20^ and increases the C input to soil C pools while reducing their rate of decomposition, leading to accumulation of soil C stocks. The increased soil C input is due to increases in leaf production and the leaf turnover rate, while the rate of soil organic matter decomposition falls because of reductions in microbial biomass C or activity^21,22^, displaying changes in the chemical composition of the soil organic matter^23^. As a result, both heterotrophic and root respiration often decline following N fertilization^20,24,25^.

Despite a relatively good understanding of the factors controlling C–N responses, the coupled responses of soil N dynamics and ecosystem C sequestration are poorly understood. N fertilization increases plant-available N fluxes of ammonium and nitrate, but only a small fraction of the added N (approximately 10% in boreal forests and 16–32% in temperate forests) is taken up by trees; the majority is immediately immobilized and locked up in soils^8,9^. Also, under conditions that favor nitrification, some of the added N is nitrified and quickly leaches out from the soil systems. These dynamics of soil N fluxes interact strongly with C dynamics, particularly in N-limited ecosystems. Consequently, there is a need to clarify the responses of soil N dynamics to N additions and to identify factors determining how ecosystem C sequestration responds to added N.

The aim of the present study was to evaluate the effects of N fertilization on C sequestration in a *Pinus densiflora* forest in South Korea*.* The average foliar N concentration of South Korean *P. densiflora* forests is about 1–1.5%^26^, thus potentially exhibiting a promising growth response to N fertilization^15^. Although *P. densiflora* forests account for as much as 25% of South Korea’s total forests (covering an area of 1.5 million hectares) and are important for both timber production and as ecosystem service providers, there are no published experimental studies on their N fertilization responses^27^. This has hampered the development of evidence-based guidelines, policies, and decision-making processes for sustainable forest management.

We conducted our study in a temperate *P. densiflora* forest in which the foliar N concentration was at 0.9% and the annual volume growth was approximately 8 m^3^ ha^−1^, indicating poor nutrition and productivity (Table 1). The annual wet N deposition in this experimental stand was quantified to 1.07 g N m^−2^ year^−1^^28^. We monitored ecosystem C and N dynamics during a 4-year period including 3 years with annual nutrient fertilization, and then revisited the site 11 years after initiating the experiment. The fertilizer employed was either NPK or PK fertilizer. We predict that if the forest is N-saturated, NPK fertilization may cause N leaching that leads to soil acidification without promoting tree growth^29^. We therefore tested PK fertilization to determine whether the studied forest is primarily limited by PK under the N-saturated condition. We hypothesized that ecosystem C sequestration is strongly responsive to annual NPK fertilization but not to PK fertilization. This has four predictions (H1–H4):

1. NPK fertilization should increase soil mineral N fluxes
2. NPK fertilization should increase foliar N concentrations and net primary production of trees, and thus increase biomass C stocks
3. NPK fertilization should increase litterfall but reduce heterotrophic respiration, and thus increase soil C stocks
4. PK fertilization should not have either of the above effects

**Table 1:** Initial stand structure in 2010: stand density, quadratic mean diameter at 1.2 m (D), basal area, standing stem volume (overbark), and soil properties at a depth of 15 cm.

Treatment	Control	P_4_K_1_	N_3_P_4_K_1_	Standard error
Stand density (trees ha^−1^)	1217	1150	1150	161
D (cm)	16.0	16.8	16.3	1.4
Basal area (m^2^ ha^−1^)	23.9	23.8	22.7	2.5
Standing volume (m^3^ ha^−1^)	134.5	135.9	129.0	15.9
Soil organic C (%)	2.40	2.66	2.81	0.33
Soil N (%)	0.74	0.84	0.92	0.11
Soil C/N ratio	32.7	32.1	31.0	1.58
Soil available-P (mg kg^−1^)	3.9	5.8	6.5	1.03

Estimates of the mean and standard error of the mean were obtained using a linear mixed model (Eq. 2; n = 6).

## Results

### Response of soil environment to nutrient fertilizations

**Figure 1:**
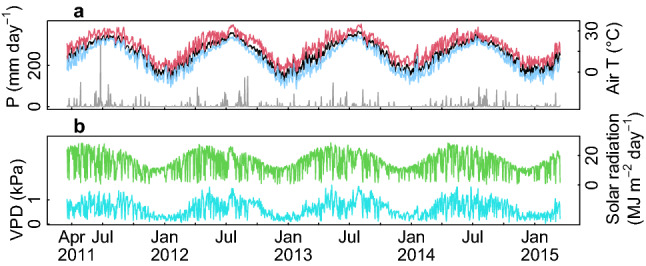
Environmental variables measured in the study forest. (**a**) Daily precipitation (grey bar) and daily mean (black), maximum (red), and minimum (blue) temperatures. (**b**) vapor pressure deficit (kPa; cyan), estimated using minimum and maximum daily temperatures during the daytime (09:00–17:00) and incident solar radiation over the canopy (green).

**Figure 2:**
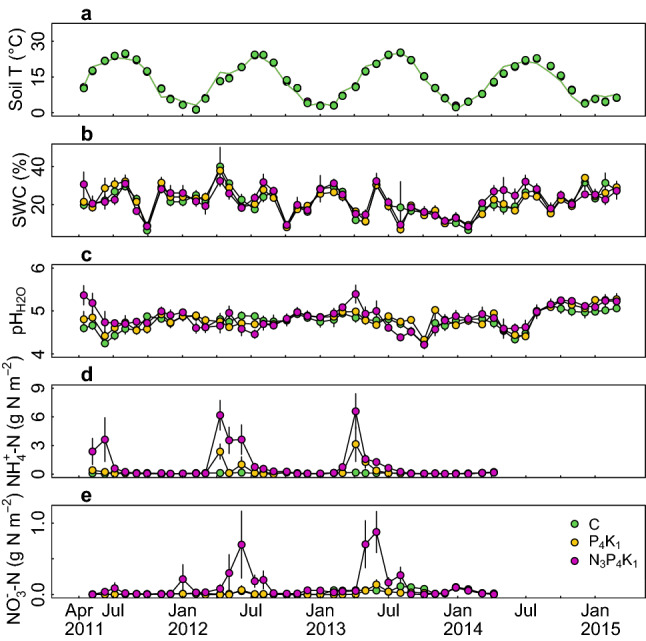
(**a**) Soil temperature at a depth of 8 cm (Soil T) according to monthly measurements (circles) and the output of a model based on the corresponding hourly air temperature (lines); no effect of treatment was observed, so the symbols and lines for different treatments overlap. (**b**–**e**) Monthly values of soil chemical properties in the 0–5 cm soil layers of each treatment (C, control; P_4_K_1_, phosphorus + potassium fertilization; N_3_P_4_K_1_, nitrogen + P_4_K_1_ fertilization): (**b**) gravimetric soil water content (SWC, %), (**c**) soil pH, (**d**) ammonium-N (NH_4_^+^-N), and (**e**) nitrate-N (NO_3_^−^-N) contents. Error bars indicate standard errors of the mean estimates (n = 6). Fertilization was applied in April 2011, April 2012, and March 2013.

**Figure 3:**
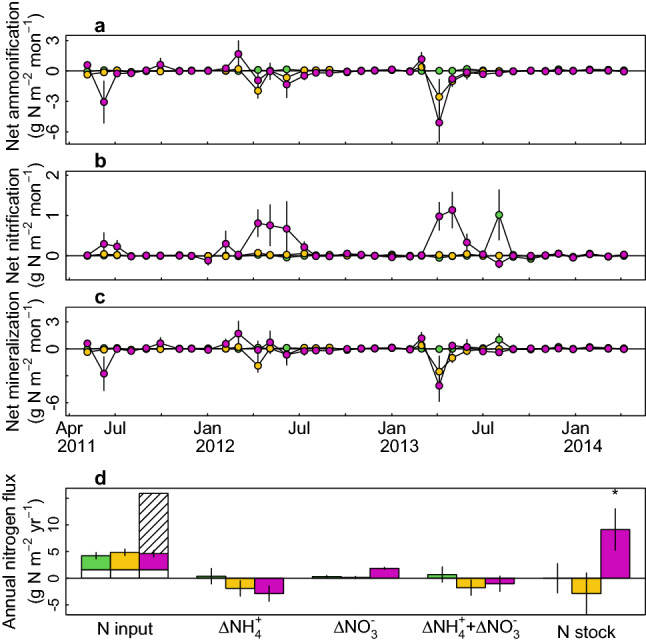
Estimates of N fluxes and pools: (**a**) monthly net ammonification, (**b**) net nitrification, and (**c**) net mineralization, calculated as the sum of the above two variables. (**d**) Annual fluxes of N input via wet-deposition (open bars), litterfall (filled bars), and fertilization (hatched bar); net ammonification (∆NH_4_^+^); net nitrification (∆NO_3_^−^); and their sum (net mineralization; ∆NH_4_^+^ + ∆NO_3_^−^); and the difference between the soil N stock in the treatment plots and that in the controls. Error bars are standard errors of the mean estimates (n = 6). Asterisks indicate estimates differing significantly from 0 (**d**, *p* < 0.05).

Variations of climate factors were greater within a year than those between years, displaying a typical climate of the region: dry and cool in spring and autumn, and wet and warm summer (Fig. 1). The fertilization treatments caused no changes in soil temperature or soil water content. Although soil pH was increased shortly after the NPK fertilization, the response diminished immediately recording no change of soil pH during the monitoring period (Fig. 2a–c; minimum *p* = 0.2 for soil pH). The treatments affected soil N fluxes and the available P concentration (Fig. 2d,e; Table S1). NPK fertilization caused immediate increases in ammonium and nitrate contents (*p* < 0.001 for both). The annual net immobilization and nitrification rates in NPK plots were −3.3 ± 1.4 g N m^−2^ year^−1^ (mean ± s.e.m., *p* = 0.04) and 1.5 ± 3.3 g N m^−2^ year^−1^ (*p* < 0.001) higher, respectively, than in control plots (Fig. 3; Table S2). PK fertilization only increased the ammonium flux, but the magnitude and duration of this increase were both less pronounced than in the NPK treatment, so no increase in the annual N flux was seen. Four years after the first fertilization (September 2014), the soil N stock in NPK plots at a depth of 0–5 cm was 27.4 ± 11.7 g N m^−2^ larger than in control plots (Fig. 3d; *p* = 0.02). The expanded soil N stock accounted for 80.8 ± 33.6% of the added N (33.9 g N m^−2^).

### Responses of ecosystem carbon pools and fluxes to the fertilization treatments

**Figure 4:**
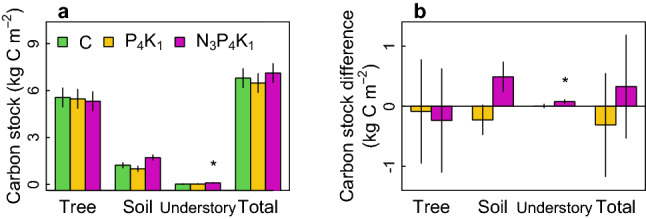
(**a**) Carbon stocks and (**b**) their differences from the control plot in 2014, 4 years after the first application of fertilizations. Soil carbon stock includes organic layer and mineral 0–15 cm. Error bars are standard errors of the mean estimates based on a mixed effect model (Eq. 2). Asterisks indicate estimated means differing (*p* < 0.05) from those for other treatments (**a**) or from 0 (**b**).

**Figure 5:**
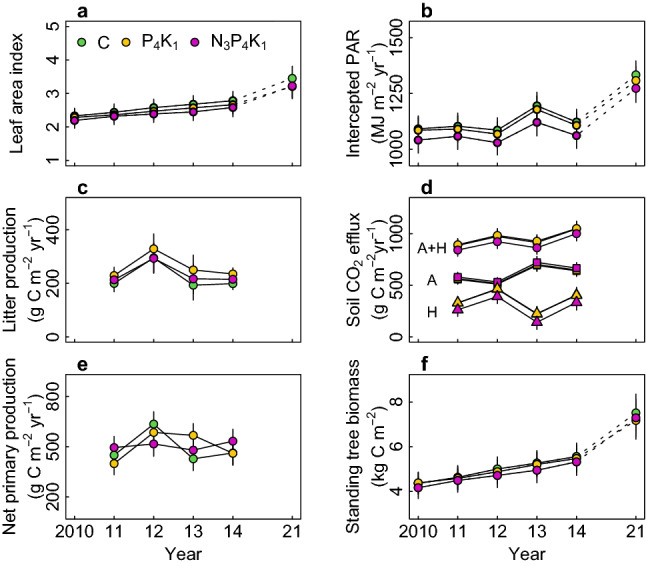
Annual dynamics of (**a**) leaf area index, (**b**) intercepted photosynthetically active radiation (PAR), (**c**) annual aboveground litter production, (**d**) soil CO_2_ efflux, estimated using Eq. (1) (A, autotrophic-; H, heterotrophic respiration), (**e**) net primary production (NPP) excluding fine root NPP, and (**f**) standing tree biomass. Error bars are standard errors of the mean estimates based on a mixed effect model (Eq. 2).

Three years of fertilization had no effect on the ecosystem C stock or flux components other than understory woody and herbaceous vegetation biomass: in the NPK plots, the C stock in understory vegetation biomass was 5.3 ± 1.3 times greater than in control plots (93.9 ± 22.3 *vs.* 17.9 ± 22.3 g C m^−2^; Fig. 4). No corresponding difference was observed between PK plots and controls. The additional 76.1 ± 31.6 g C m^−2^ in understory biomass, however, accounted only for 1.4% of the total vegetation biomass C stock (5.6 ± 0.6 kg C m^−2^) of control plots, being trivial to record a greater ecosystem C accumulation. No response to the treatments was observed for other variables associated with canopy C assimilation (i.e., the leaf area index and canopy-intercepted photosynthetically active radiation; Fig. 5a,b) or for variables associated with C fluxes including the net primary production of trees (NPP, excluding fine roots), litterfall, and root and heterotrophic respiration (maximum *p* = 0.1 for soil CO_2_ efflux; Fig. 5c–e). Consequently, the ecosystem C stock did not differ between treatments (*p* = 0.8 for the total ecosystem C stock, minimum *p* = 0.06 for the soil C stock; Fig. 5f). This lack of response was consistent with the fact that there were no detectable differences between treatments with respect to (1) the litter decomposition rate over 4 years, (2) the monthly monitored increment of the focal trees, or (3) the results of disc ring analyses performed using samples collected 11 years after initiating the experiment (October 2021; Fig. S2). In addition, no tree mortality was observed in the studied plots over 12 years. Across the treatment plots, the annual stem volume increment amounted to 9.7 ± 2.1 m^3^ ha^−1^ year^−1^, the NPP to 501.1 ± 70.0 g C m^−2^ year^−1^, annual CO_2_ efflux to 943 ± 66 g C m^−2^ year^−1^, and heterotrophic respiration to 612 ± 51 g C m^−2^ year^−1^.

## Discussion

Increased ecosystem C sequestration is a general response to N fertilization^30,31^. A recent meta-analysis^30^ indicated that tree growth commonly responds to N addition in temperate and boreal forests, particularly in locations where biomass productivity is low (NPP of aboveground woody biomass ≤ 300 g C m^−2^ year^−1^) and the N deposition rate is below 1.5 g m^−2^ year^−1^. The forest examined in our work^28^ had a productivity of 275 ± 40 g C m^−2^ year^−1^ and a wet N deposition rate of 1.07 g m^−2^ year^−1^, so a positive growth response to N fertilization was expected. Moreover, annual mean net N mineralization rate in the native condition amounted to 1.0 g N m^−2^ year^−1^, located in the lower end of the mineralization rates across temperate forests^32^. Applying the estimate of N mineralization rate to the relationship between the aboveground NPP and the annual N mineralization rate (ANPP = 404 + 53 × mineralized N)^32^, the aboveground NPP was predicted to be 457 g C m^−2^ year^−1^, in good agreement with our estimate, 465 ± 54 g C m^−2^ year^−1^. This validates our estimates of annual mineralization rates and indicates a lower soil N availability and biomass productivity in our study forest. Despite increased soil N fluxes by NPK fertilization (supporting H1; Fig. 2d,e), NPK fertilization affected neither the foliar N content nor the productivity of any biomass compartment, indicating that the biomass stock was unresponsive to the fertilization (refuting H2; Fig. 5). Similarly, no response to NPK treatment was observed for any of the variables used to characterize soil C dynamics—soil respiration, litter production, and litter decomposition—so there was no evidence of greater accumulation of soil C in NPK plots (refuting H3; Fig. 4). No effect of PK fertilization on the above variables (supporting H4), and unchanged soil acidity with the high N recovery in the NPK soil indicate that the studied forest was not N-saturated^1,2,5,31^.

We acknowledge that our study setting is limited for quantification of ecosystem C–N response to N fertilization, because trees at the edges can extend the rooting area beyond the treatment-plot (100 m^2^) and fertilized N can be diluted over the buffer zone. Nevertheless, if the fertilization *per se* was effective on trees at all, it should have been visible of tree growth and/or foliar nutrition at stand-scale, individual trees, or at least the intensively monitored focal trees within the center of the plots, based on the following reasoning. First, our NPK treatment resulted in increased mineral N fluxes and soil N stock, validating the efficacy of the treatments on soil N availability. Second, roots of pine trees can take up 90% of soil nutrients within a 3 m distance and the uptake distance becomes even shorter when nutrients are added^33,34^. So the trees in the center of the plots must have been responsive. Lastly, other highly profiled studies at a small scale of forest ecosystem ranging a 1.1 to 26 m^2^ plot showed significant C responses to manipulations of the environmental conditions (CO_2_, air- and soil temperatures, and water availabilities)^35–37^, even if nutrient fertilization to a nutrient-deficient forest is considered to show even greater impact on C dynamics than elevated CO_2_ or warmings^38–40^. We, therefore, conclude that the fertilization did not lead to shifted C dynamics of our N-poor forest regardless of the treatment scale. We discuss its potential mechanisms in the coming paragraphs.

It has been suggested that increasing soil N content by N fertilization can cause foliar nutrient imbalances that ultimately reduce forest growth and increase the risk of mortality due to pests and pathogens^10–14^. As we observed no increase in foliar N levels or tree mortality, this mechanism did not appear to be active in the studied forest. Another possible adverse consequence of N fertilization is N-induced soil acidification, leading to reduced P and base saturation and depleting the soil’s Ca content^41^. Again, our results suggest that this mechanism was not important in the studied forest. Our fertilizer applied N in a form of urea, which rather increases pH and only causes soil acidification when leaching of the nitrified nitrate-N occurs. NPK fertilization immediately increased pH, indeed, but the monthly soil pH measurements did not differ appreciably between treatment plots over 4 years (Fig. 2c). Moreover, most of the added N was recovered in the soil and soil pH did not decline, suggesting minimal N leaching. We note that the soil pH was low (4.2) in all cases, irrespective of the applied fertilization regime, which may inhibit root uptake of available N to some extent^41^.

We analyzed the variables affecting aboveground and belowground C dynamics using a classical framework^18,42^ (Fig. 5) in which the N response of biomass production results from (1) increased foliar N levels and/or increasing light harvesting by the canopy, leading to increased gross primary production^7,43–45^ and (2) a shift of C partitioning from below- to aboveground biomass^17,46,47^. However, none of these responses were observed in our forest despite the site’s obvious N deficiency (0.9% foliar [N]), poor site-specific N deposition rate, and low biomass productivity. We therefore conclude that the N response of the studied forest was limited in the first instance by the rate at which the trees could acquire N.

Root N acquisition is determined by soil N availability, the plant’s N requirements, and the capacity for delivering available N in the soil solution to the root surface through the soil matrix^48^. N transport in the soil is therefore restricted by soil moisture, adsorption, and microbial interactions^49^. Given the observed improvement in soil N fluxes following NPK fertilization and the poor foliar nutrition of the trees in our study plots, the supply of N, both less mobile ammonium and mobile nitrate in the soil matrix, should have been sufficient to meet trees' N demand. However, NPK fertilization did not improve the N nutrition of the trees. We argue that three factors mainly responsible for the non-responsiveness: immobilization of the added N, competitions with sub-canopy vegetation, and interaction with water availability. First, ammonium, less mobile chemical form in the soil, can quickly bind to soil matrix by the ion exchange complex as well as being immobilized by microbes before trees taking up, as supported by a high immobilization rate and increased soil N stock (Fig. 3). Second, despite a large N immobilization rate, increased ammonium flux was maintained for a few months after the fertilization. Nevertheless, improved trees’ N nutrition was not recorded. We speculate that understory vegetation outcompeted the canopy trees for the ammonium resource, as supported by increased growth of understory vegetation. Third, the soil’s dryness may have reduced its capacity for N delivery, in particular of nitrate, and trees’ uptake capacity of both mineral N sources over microbes^49^. Indeed, volumetric water content was 20.4 ± 0.9% (temporal variation, n = 48 months; 21.7 ± 1.0% for gravimetric), recharging only approximately 20% of the capacity of available soil water. The dryness of the soil may also have limited the trees’ transpiration capacity, particularly during spring when most foliage growth occurs. Transpiration drives soil mass flow, i.e. the bulk movement of water in the soil, which can greatly increase trees’ ability to compete with microbes for acquisition of soil N^49^. For example, in a nutrient-poor boreal forest where water was not limiting, N uptake increased with trees’ water uptake and transpiration under fertilized conditions^33^. Moreover, interactions between water availability and N fertilization have been reported across temperate and boreal regions, showing that tree growth did not respond to fertilization under conditions of soil water deficit^50^.

Whether due to impaired root N uptake or a high microbial capacity for N immobilization, the high N fluxes induced by NPK fertilization were immobilized (Fig. 3). This may be linked to the fact that the soil C stock in the NPK plots increased by 42.3 ± 20.9% (Fig. 4; *p* = 0.06): an increased input of labile organic compounds from fine root turnover and fresh litter from understory vegetation could trigger an increase in microbial N immobilization^51^. Indeed, a recent meta-analysis showed that organic C additions increased microbial N immobilization by 105% when compared to unamended soils^52^. However, the positive effect of N fertilization on N immobilization may be offset by a reduction in total microbial biomass following N fertilization: a recent study showed that high levels of N addition (> 10 g N m^−2^ year^−1^) can cause strong reductions in microbial biomass C^21^. The soil N stock was significantly larger in NPK plots than in PK or control plots, amounting to 80% of the added N (Fig. 3d). The unrecovered 20% of added N was presumably diluted vertically over untreated plots via litter movements or lost via denitrification and ammonium volatilization yet to a lesser extent due to the lower pH and dry soil condition^53^.

We have presented several lines of evidence indicating that N fertilization was ineffective at increasing ecosystem C sequestration in the studied forest. However, additional data and manipulative experiments are needed to elucidate the mechanisms responsible for this outcome. For example, we lack data on microbial C and N stocks, as well as adequate information on the variation in the soil water content over the study period. These limitations make it difficult to quantify and characterize the role of soil microbes in soil N dynamics, because the contribution of soil microbes depends on both the soil water content and the native soil fertility^51,54^. Inter-annual response of C flux components to changes in soil water availability may have been partially masked by the high seasonal variability of climate factors and their interactions at the studied site (Fig. 1)—dry and cool spring and autumn, and wet and warm summer. We also point that our study design cannot rule out the possibility of soil macro-(e.g. Ca and Mg) or some micro-nutrients primarily limiting the growth. Although irrigation and lime fertilization are very uncommon forestry practices in South Korea, further manipulative studies examining the effects of water availability and nutrient uptake are needed to obtain a deeper mechanistic understanding of C and N cycling following different management interventions.

Demands for forest ecosystem services including C sequestration and timber production have increased in recent years. Sustainable forest management practices are needed to satisfy these different demands without having to trade one off against another. Fertilization is one of the most common practices in the forestry sector and is frequently applied over a period of multiple years, although even single fertilizer applications have been reported to be powerful tools for increasing wood production and ecosystem C sequestration in nutrient-limited forests. However, no such response was observed in our study despite repeated fertilization over 3 years. This means that N fertilization does not guarantee increased C sequestration or biomass growth and should therefore be applied with caution even for forests with a poor N nutrition.

## Methods

### Setting

This study was conducted in approximately 40-year-old naturally regenerated *P. densiflora* stands in Wola National Experimental Forest in Gyeongnam province in South Korea (35°12′ N, 128°10′ E; Table 1). The productivity of this forest is low, with a dominant tree height of 10 m at 20 years of age. Over the last 10 years, the mean annual precipitation was 1490 mm, of which one third fell during summer (July–August), and the mean temperature was 13.1 °C. The vegetation growing season generally lasts for approximately 200 days, extending from early April to October. The soil texture is a silt loam originating from sandstone and shale (clay 13.0 ± 0.8%, silt 44.1 ± 1.3%, sand 42.9 ± 1.6%; n = 18). The given texture results in volumetric water contents at 13.4 ± 0.7% (m^3^ m^−3^) at permanent wilting point (1500 kPa) and 40.7 ± 1.2% at field capacity (10 kPa)^55^. The understory is covered with *Lespedeza* spp., *Quercus variabilis*, *Q. serrata*, *Smilax china*, and *Lindera glauca*.

In 2010, we selected two adjacent *P. densiflora* stands approximately 100 m apart from each other (180 m and 195 m above sea level, on slopes of 15° and 33°, both stands face south). Following a completely randomized design, we established nine plots (10 × 10 m^2^ with a 5 m untreated buffer) within each stand, of which three were randomly assigned to annual NPK fertilization, three to PK fertilization, and the rest to a control treatment without fertilization. The fertilizer, composed of urea, fused superphosphate and potassium chloride (N_3_P_4_K_1_) or P_4_K_1_ was added manually by deposition on the forest floor for 3 years in April 2011, April 2012, and March 2013. Over these 3 years, the NPK plots received 33.9 g N, 45 g P, and 11.1 g K m^−2^, while the PK plots received 45 g P and 11.1 g K m^−2^.

### Tree and stand measurements

The standing biomass of trees was estimated using a combination of site-specific allometric equations based on destructive harvesting^56^ and repeated measurements of the dimensions of all trees in each plot (5–18 trees plot^−1^). The stem diameter at 1.2 m (D) was measured for all trees in each plot for which D was ≥ 6 cm. Selecting a representative tree in size for each plot within the 4 × 4 m^2^ center of the plot, we measured the tree height (H) and crown base for the representative trees. Measurements were performed in April and September 2011, September 2012–2014, and October 2021. We observed no effect of fertilization on the relationship between D and H or between D and crown base, so we assumed no effect on the allometric functions for foliage or branch biomass. A dendrometer band (Series 5 Manual Band, Forestry Suppliers Inc., Jackson, MS, USA) was installed on 18 representative trees (one per plot) to monitor radial growth monthly.

Three 0.25 m^2^ circular litter traps were installed 60 cm above the forest floor in each plot in April 2011. Litter was collected at 3-month intervals between June 2011 and March 2015. The litter from each trap was transported to the laboratory and then oven-dried at 65 °C for 48 h. All dried samples were separated into needles, bark, cones, branches, and miscellaneous components, and weighed separately.

In September 2014, we estimated the biomass of understory vegetation, separately for woody plants and herbaceous plants. All woody plants < 1.2 m tall in each plot were harvested, while herbaceous plants were harvested within three 1 × 1 m^2^ subplots within each plot. The samples were separated into stems, branches, and leaves, and then oven-dried at 65 °C and weighed.

The leaf area index (LAI) was estimated for each treatment as the product of the specific leaf area (cm^2^ g^−1^) and the sum of the standing leaf biomass and annual foliage litterfall^57^. As most litterfall occurred after summer, adding the foliage litterfall to the total foliage mass is necessary to accurately estimate the LAI during the summer season. The estimated LAI and recorded annual photosynthetically active radiation (PAR) were used to estimate the canopy-intercepted PAR using the Beer-Lambert light extinction function [intercepted PAR = PAR·(1–*e*^−*k*·*LAI*^)] with an extinction coefficient (*k*) of 0.52^58^.

### Estimation of stocks and fluxes of soil carbon and nitrogen

Annual fluxes of C and N were estimated from the measured monthly fluxes. Biomass stocks were estimated annually, while soil stocks were estimated only at the end of the investigation.

Soil C fluxes were characterized by determining the total soil CO_2_ efflux and heterotrophic respiration, whereas soil N fluxes were characterized by determining the net mineralization and nitrification rates using an in situ incubation method. Total soil CO_2_ efflux was separated into root- and heterotrophic respiration using a trench method^59^. A root exclusion barrier (50 cm diameter, 30 cm depth, PVC) was installed in each plot, 4 weeks before the treatments were initiated. Two soil collars (10 cm diameter, 5 cm depth, PVC) were installed inside the rooting barrier to measure heterotrophic respiration and another two were installed outside the rooting barrier to measure heterotrophic + root respiration. Vegetation was removed within the rooting barriers but litterfall was kept. Soil CO_2_ efflux was measured once a month using an infrared gas analyzer (Model EGM-4, PP-Systems, Hitchin, Hertfordshire, UK) equipped with a flow-through closed chamber (Model SRC-2). Measurements were performed between 10:00 and 13:00 over the study period. Soil temperature was also measured at a depth of 8 cm near the soil CO_2_ efflux collar using a digital soil temperature probe (K-type, Summit SDT 200, Seoul, Korea).

On each day when the soil CO_2_ efflux was measured, two soil cores were collected from each plot at a depth of 5 cm using a 100 cm^3^ core soil sampler. One sample was placed in a plastic bag and the other was returned to the soil and incubated to estimate the net N mineralization rate. We thus collected two soil samples from each plot every month, one fresh and one incubated. Both samples were transported to the laboratory and their fresh weight was measured. A 10 g portion of the fresh soil sample was oven-dried for 48 h at 105 °C to quantify the soil’s gravimetric water content and the rest was kept to determine the concentrations of nitrate and ammonium and the soil pH (measured using a 1:5 soil water suspension with an ion-selective glass electrode; Istec Model pH-220L, Seoul, Korea). The bulk density of each soil sample was calculated from its gravimetric water content and fresh weight.

Ammonium and nitrate were extracted from soil samples (5 g) using 50 ml of a 2 M KCl solution in a mechanical vacuum extractor (Model 24VE, SampleTek, Science Hill, KY, USA). The resulting solutions were then immediately placed in a cooler at 4 °C for storage. The ammonium and nitrate concentrations of the solutions were determined using an auto analyzer (AQ2 Discrete Analyzer, Southampton, UK). The mineral N concentration was measured throughout the period when fertilizer was applied, from April 2011 to April 2014. The net rates of ammonification and nitrification were estimated based on the differences in the ammonium and nitrate contents of the soil, respectively, between before and after the incubation. The sum of these two rates was taken as the net mineralization rate. If the net mineralization rate was negative, it was regarded as a net immobilization rate.

Additional soil samples were collected from four randomly selected points in each plot at depths of 0–15 cm both 4 weeks before starting the treatments and at the end of the investigation (2014). Samples were pooled within a plot and their concentrations of C, N, and available P were determined to capture the soil’s initial condition and the treatment response. C and N were analyzed with an elemental analyzer (vario Macro cube, Elementar Analysensysteme GmbH, Germany). The available P concentration was determined by extraction using NH_4_F and HCl solutions^60^ and analyzed using a UV spectrophotometer (Jenway 6505, Staffordshire, UK). C and N stocks were estimated by measuring the concentrations of both elements in a 100 cm^3^ core and dividing by the bulk density of the soil in the 0–5 cm layer.

### Data analysis

The trees’ NPP was estimated as the product of the annual biomass increment, woody components of litterfall, and the C content of each biomass component^56^. Foliage NPP was estimated as the sum of the annual leaf litterfall and the annual foliage biomass increment, which in turn was estimated using the relevant allometric equation. Total soil CO_2_ efflux and heterotrophic respiration were modelled as exponential functions of the soil temperature and moisture content in each treatment:

1 $${\text{ln }}\left( {R_{S \, ijkl} } \right) = b_{0} + b_{1} \times T_{S\,ijkl} + b_{2} \times F_{j} + \, b_{3} \times (T_{S} \times F)_{ijkl} + b_{4} \times S_{k} + \epsilon_{ijkl}$$

Here, *R*_*Sijkl*_ is the *l*th observation of the total soil CO_2_ efflux or heterotrophic respiration in replicated plot *i* (*i* = 1–3) of treatment *j* (*F*, *j* = control, PK, NPK fertilizations) within stand *k* (*S*, *k* = 1, 2). *T*_*S*_ is the soil temperature (°C) and *T*_*S*_ × *F* is the interaction term. The *b*s are coefficients to be estimated and *ε* represents the final residuals. After finding no effect of soil moisture content on the temperature sensitivity of *R*_*S*_ (*b*_*1*_) or base *R*_*S*_ (*b*_*0*_) (*p* = 0.3 for total CO_2_ efflux and 0.5 for heterotrophic respiration; Table S3), only the soil temperature and plot-stand factors were included in the final model. The models included both terms separately for each year to enable propagation of spatiotemporal variation in the annual estimates within a treatment. Because we only measured the soil temperature manually once a month, hourly estimates of the soil temperature were obtained by deriving a relationship between the soil temperature and the corresponding air temperature (Fig. 2a; *R*^2^ = 0.954, relationship unaffected by treatment, *p* = 0.99). Using the above model and the air temperature, we estimated the annual soil CO_2_ efflux and heterotrophic respiration.

The effects of the fertilization treatments on above- and belowground variables were determined using a mixed effect ANOVA model to account for the completely randomized design used in the adjacent two stands. The stand term was treated as a random effect and the fertilization treatments were treated as fixed effects.

2 $$Response_{ijkl} = \mu + F_{j} + Y_{l} + (F \times Y)_{jl} + S_{k} + \epsilon_{ijkl}$$

Here, *Response*_*ijkl*_ is a response variable in replicated plot *i* of treatment *j* within stand *k* in year *l* (*l* = 1–4).  *µ* is the grand population mean, *F* is the fixed effect of the treatment, *Y* is the fixed effect of the year as a dummy, and *S* is the random effect associated with stand. For the monthly soil N mineralization and nitrification models, we replaced year *Y* with month (*l* = 1–36).

All statistical analyses and parameter estimations for the devised models were performed using R (v. 4.1.3): the *lm* function was used to devise linear relationships and the *lme* function of the nlme package was used to determine the coefficients of the mixed effect ANOVA model (Eq. 2). Means were compared and separated using Tukey's test with a significance threshold of *p* < 0.05, which was performed using the *lsmeans* and *cld* functions of the lsmeans package. The final residuals (*ε*_*ijkl*_) were normally distributed, which was confirmed visually by plotting them against predictions.

### Ethical approval

The present study conducted in the Wola National Experimental Forest on *P. densiflora* trees, including the collection of plant/soil materials, complied with local (Gyeongsangnam-Do) and national (South Korea) guidelines and legislation, with the permission from the administering institute, National Institute of Forest Science.

## Supplementary Information

Supplementary Information.

## Data Availability

All data used during the present study are available from the corresponding author on request.
