# Supplementary material for: No impact of nitrogen fertilization on carbon sequestration in a temperate Pinus densiflora forest
Source: Sci Rep. 2023 Mar 6;13:1743. doi: 10.1038/s41598-023-27989-3 (PMC9988963; doi:10.1038/s41598-023-27989-3)
Supplement: Supplementary file 1 — Supplementary Information. [file 41598_2023_27989_MOESM1_ESM.docx]

**No impact of nitrogen fertilization on carbon sequestration in a temperate *Pinus densiflora* forest**

Gyeongwon Baek^1,+^ , Hyungwoo Lim^2,3,+^, Nam Jin Noh^4^, and Choonsig Kim^1,*^

^1^Division of Environmental and Forest Science, Gyeongsang National University, Jinju 52725, South Korea

^2^Department of Forest Ecology and Management, Swedish University of Agricultural Sciences (SLU), SE-901 83, Umeå, Sweden

^3^Institute of Ecology and Earth Sciences, University of Tartu, 50409, Tartu, Estonia

^4^Department of Forest Resources, Kangwon National University, Chuncheon 24341, South Korea

^*^Corresponding. ckim@gnu.ac.kr

^+^these authors contributed equally to this work

**Table S1.** Concentration of total nitrogen and available phosphorus in mineral soil (0 – 15 cm) from the study stands before and four years after initiating the fertilization treatments.

| Treatment | Total N (mg g^–1^) | | Available P (mg kg^–1^) | |
| --- | --- | --- | --- | --- |
|  | 2010 | 2014 | 2010 | 2014 |
| Control | 0.7 ± 0.1a | 1.5 ± 0.2ab | 3.9 ± 0.4a | 5.4 ± 0.67b |
| P_4_K_1_ | 0.8 ± 0.1a | 1.3 + 0.1b | 5.8 ± 1.6a | 47.5 ± 20.7ab |
| N_3_P_4_K_1_ | 0.9 ± 0.0a | 2.1 ± 0.3a | 6.5 ± 0.7a | 105.7 ± 23.5a |

The error term is the standard error of the mean estimate (n = 6). Different letters indicate significant differences between treatments (*P* < 0.05).

**Table S2**. Estimated annual nitrogen (N) flux (g N m^-2^ yr^-1^) and stock (g N m^-2^).

| Year | Treatment | ^1^Internal N input | ^2^External N input | ^3^Net ammonification | Net nitrification | ^3^Net mineralization | Soil N stock |
| --- | --- | --- | --- | --- | --- | --- | --- |
| 2011 | Control | 2.15 | 1.6 | 0.10 | 0.00 | 0.10 |  |
|  | P_4_K_1_ | 2.66 | 1.6 | -0.28 | 0.06 | -0.22 |  |
|  | N_3_P_4_K_1_ | 2.48 | 1.6+11.3 | -0.34 | 0.74 | 0.41 |  |
| 2012 | Control | 3.48 | 1.6 | 0.52 | 0.05 | 0.57 |  |
|  | P_4_K_1_ | 4.14 | 1.6 | -1.89 | 0.24 | -1.65 |  |
|  | N_3_P_4_K_1_ | 3.80 | 1.6+11.3 | -1.90 | 2.47 | 0.58 |  |
| 2013 | Control | 2.25 | 1.6 | 0.54 | 0.90 | 1.43 | 68.42 |
|  | P_4_K_1_ | 2.97 | 1.6 | -3.57 | 0.13 | -3.44 | 59.75 |
|  | N_3_P_4_K_1_ | 2.73 | 1.6+11.3 | -6.40 | 2.33 | -4.07 | 95.81 |
| Degree of freedom | | 44 |  | 44 | 44 | 44 | 14 |
| SE of the model estimate | | 0.65 |  | 1.81 | 0.40 | 1.79 | 8.28 |
| treatment | | **0.027** |  | **0.024** | **<0.001** | 0.135 | **0.021** |
| year | | **<0.001** |  | **0.044** | **0.032** | 0.187 | NA |
| treatment x year | | 0.993 |  | 0.255 | 0.139 | 0.283 | NA |

^1^Internal N input was estimated based on the N contents of annual litterfall; ^2^external N input is wet N deposition via throughfall estimated in the stands (1.6 g N m^-2^ yr^-1^, no effect of fertilization; Kim et al. 2017) and added N (11.3 g N m^-2^ yr^-1^); ^3^negative values indicate net nitrogen immobilization.

| Year | DF | b_1_ | p-value | b_0_ | p-value | b_2_ | b_3_ |
| --- | --- | --- | --- | --- | --- | --- | --- |
| Total soil CO_2_ efflux (soil CO_2_ efflux outside the root barriers) | | | | | | | |
| 2011 | 226 | 0.076 (0.006) | p<.001 | -0.095 (0.156) | 0.542 | 0.843 | 0.712 |
| 2012 | 198 | 0.063 (0.007) | p<.001 | 0.333 (0.142) | 0.020 | 0.346 | 0.219 |
| 2013 | 220 | 0.071 (0.005) | p<.001 | -0.006 (0.141) | 0.965 | 0.735 | 0.820 |
| 2014 | 234 | 0.090 (0.009) | p<.001 | -0.116 (0.16) | 0.471 | 0.296 | 0.951 |
| Heterotrophic respiration (soil CO_2_ efflux within the root barriers) | | | | | | | |
| 2011 | 226 | 0.057 (0.007) | p<.001 | -0.013 (0.152) | 0.934 | 0.124 | 0.306 |
| 2012 | 198 | 0.059 (0.007) | p<.001 | -0.088 (0.184) | 0.633 | 0.404 | 0.310 |
| 2013 | 220 | 0.039 (0.007) | p<.001 | 0.414 (0.171) | 0.016 | 0.921 | 0.747 |
| 2014 | 234 | 0.081 (0.008) | p<.001 | -0.270 (0.157) | 0.087 | 0.305 | 0.575 |

**Table S3.** Parameter estimates for the soil CO_2_ efflux model (Eq. 1).

Eq 1: ln (*R_S ijkl_*) = *b_0_* + *b_1_⋅T_S ijkl_* + *b_2_⋅F_j_ + b_3_*⋅(*T_S_⋅F*)*_ijkl_* + *b_4_*⋅*S_k_* + *ε_ijkl_*

**Figure S1**. Soil CO_2_ efflux and heterotrophic respiration in relation to soil temperature at 8 cm for the years 2011 to 2014 separately (a-d, 2011 – 2014, respectively). Circles indicate total soil CO_2_ efflux (root + heterotrophic respiration); triangles indicate heterotrophic respiration, determined using a root exclusion trenching method. No effect of fertilization treatments was observed on the temperature sensitivity of either total soil CO_2_ efflux (p = 0.254) or heterotrophic respiration (p = 0.515). The separate models for each year were used to estimate annual respirations; the corresponding parameter estimates are listed in Table S3.

**Figure S2. (a-c)** Focal individual tree growth analyses: **(a)** relationships between diameter and height in 2010 (circles; solid line) and 2021 (triangles; dotted line), **(b)** monthly dendrometric measurements, and **(c)** relationship between stem diameter at 1.2 m (cm) in different periods (2010 and 2014, circles; 2014 and 2021, triangles). **(d)** Mass loss (g g^-1^) of buried litterbags over the study period. Error bars are standard errors of the estimates (n = 6). The focal trees were sampled within the 4 m × 4 m center of the plots.
